# Establishment of Novel Simple Sequence Repeat Markers in *Phragmites australis* and Application in Wetlands of Nanhui Dongtan, Shanghai

**DOI:** 10.3390/biology14040356

**Published:** 2025-03-28

**Authors:** Shaozu Ma, Yifei Shen, Min Li, Ruitong Jiang, Luyi Cai, Tingting Wu, Linxi Gao, Meiqin Wu, Peimin He

**Affiliations:** 1 College of Oceanography and Ecological Science, Shanghai Ocean University, Shanghai 201306, China; a519052241@163.com (S.M.); shenyifei317@163.com (Y.S.); asharjj@outlook.com (R.J.); clyiiiiii@163.com (L.C.); m220501178@st.shou.edu.cn (T.W.); gaolinxi0110@163.com (L.G.); 2 Department of Biological Sciences, Wayne State University, Detroit, MI 48202, USA; lmin177@163.com

**Keywords:** *Phragmites australis*, SSR marker, genetic diversity, population genetic structure

## Abstract

Common reed (*Phragmites australis*) is a widely distributed perennial herb of high ecological significance in wetlands worldwide. This research developed 15 SSR markers through whole-genome sequencing and assessed the genetic diversity across four representative reed populations (DB, JPDP, YJRHK, DD) in the coastal wetlands of Nanhui Dongtan, Shanghai. A total of 113.5 alleles (*Na*) were detected, averaging 7.567 alleles per locus, with an expected heterozygosity (*He*) of 0.690 and a Shannon diversity index (*I*) of 1.485. Polymorphism information content (PIC) values (ranging from 0.577 to 0.872, average 0.770) indicated strong resolution power for discriminating subtle genetic differences across populations. Among the four populations, JPDP displayed the highest genetic diversity parameters (*Na* = 12.667; *I* = 2.020), whereas DD exhibited the lowest, consistent with intensive demographic screening under saline stress and competition from *Spartina alterniflora*. Bayesian clustering analysis (STRUCTURE) and UPGMA dendrograms further revealed that the four populations split into two major subgroups, which underlines both the influence of geographic and ecological factors on genetic structuring and the persistence of gene flow. In sum, the newly developed SSR markers demonstrated robustness and sensitivity in elucidating the overall high genetic diversity but moderate genetic differentiation of reed populations in Nanhui Dongtan. These findings not only offer insights into local adaptation mechanisms in coastal wetlands but also provide a technical basis for the conservation and utilization of *P. australis.*

## Simple Summary

Common reeds (*Phragmites australis*), vital wetland plants, face challenges from environmental changes. To understand how coastal reeds adapt, we created 15 genetic markers to study four reed groups in Shanghai’s wetlands. Results revealed high genetic diversity overall, but one group (DD) showed lower diversity, likely due to salt stress and competition from invasive *Spartina alterniflora*. The reeds split into two clusters, shaped by both geography and environmental conditions, yet maintained some genetic mixing. This work helps identify resilient reed populations, supports conservation efforts to sustain reeds in changing environments and informs strategies to protect wetlands, which are crucial for biodiversity and coastal health.

## 1. Introduction

Common reed (*Phragmites australis* (Cav.) Trin. ex Steudel) is a widely distributed perennial herbaceous plant that propagates primarily through its rhizomes, with sexual reproduction via seeds serving as a supplementary mechanism. *P. australis* is globally distributed—except in Antarctica [1]—and is commonly found along riverbanks, in deserts, saline and alkaline regions, lowlands, marshes, and coastal zones. Accordingly, it makes a significant contribution to the biodiversity of reed wetlands [2,3,4]. Owing to its remarkable adaptability and broad ecological amplitude, reeds are recognized as keystone species in wetland community formation [5,6].

In addition, reed effectively absorbs substantial quantities of nutrients such as nitrogen and phosphorus and is capable of accumulating heavy metals (e.g., Cr, Hg, Pb, Zn, and As) from its environment. This capacity plays a vital role in the purification of wetland water and the ecological restoration of saline-alkali soils [7,8]. Moreover, reed mitigates the impact of wind and waves, reduces water flow velocity, and promotes sediment deposition, thereby contributing to dike protection, shoreline stabilization, mudflat formation, and the development of coastal wetland landscapes. Notable reed-formed landscapes include those of the Danube Delta, Tianjin Qilihai, and Shanghai Chongming Dongtan [9].

However, rapid economic development and urban expansion in Shanghai have led to a progressive reduction in the areas of coastal mudflats and salt marshes [10,11]. From 1990 to 2018, mudflat areas decreased by approximately 52%, while tidal wetlands experienced a net loss of about 18% [12]. Shanghai’s Nanhui Dongtan, located along the central coast of China, has experienced severe degradation of reed populations and a decline in wetland biodiversity, largely due to the proliferation of *Spartina alterniflora* populations [13,14]. In response to these environmental challenges, the Shanghai municipal government has initiated a large-scale reed planting program in the coastal wetlands of Nanhui Dongtan. This initiative aims to substantially restore reed populations, thereby reconstructing and enhancing the diversity and stability of the wetland ecosystem [15].

Genetic diversity constitutes the fundamental basis for a species’ adaptive capacity and forms a critical component of overall biodiversity. Populations exhibiting higher genetic diversity generally demonstrate greater resilience in response to environmental stressors—such as salinity, tidal fluctuations, anthropogenic disturbances, and climate change—whereas a reduction in genetic diversity may compromise the resilience and resistance of reed populations [16]. Elevated genetic diversity enhances the potential of a genotype to withstand various biotic and abiotic challenges, thereby reinforcing the functional stability and resilience of ecosystems under adverse conditions [17,18]. For example, the molecular mechanisms underpinning reed’s salt tolerance involve multiple layers of regulation, including phenotypic plasticity, adaptive functional traits, and DNA demethylation [19,20,21]. Comparative transcriptomic analyses have demonstrated that under salt stress, transcripts associated with glutathione metabolism are upregulated in tidal reeds but not in freshwater reeds. Among these, genes encoding glutathione reductase (GR), glucose-6-phosphate 1-dehydrogenase (G6PDH), 6-phosphogluconate dehydrogenase (6PD), glutathione S-transferase (GST), and L-ascorbate peroxidase (LAP) are notably upregulated, thereby enhancing the plant’s salt tolerance. Consequently, reed populations with lower genetic diversity are more susceptible to damage and exhibit slower recovery following natural disasters or anthropogenic disturbances—a phenomenon that not only jeopardizes the survival of reeds but may also adversely affect other species dependent on it, ultimately escalating the risk of severe ecosystem degradation and species extinction [22].

A variety of methodologies have been employed to investigate genetic diversity, including the use of morphological markers, chromosomal mutation analyses, isoenzyme profiling, and molecular marker techniques [23]. Among these, DNA-based genetic markers are particularly valuable for elucidating patterns of population distribution and evolutionary dynamics [24,25]. Simple sequence repeats (SSRs), or microsatellite DNA, represent a class of molecular markers characterized by high polymorphism, co-dominant inheritance, excellent reproducibility, and stability [26,27]. Saltonstall initially developed SSR markers for *P. australis* by adapting primers from other species, and these markers have since been extensively deployed in studies examining the genetic diversity of *P. australis* [28,29,30]. Furthermore, Lambertini et al. [31] utilized AFLP to assess genetic variation in reed and to delineate its global phylogenetic structure, revealing distinct evolutionary lineages among clonal populations from South America, the Gulf Coast of the United States, and octoploid populations from East Asia and Australia. Previous studies on *P. australis* genetic diversity have predominantly focused on continental-scale phylogeography or single-stress responses [20,31]. However, few efforts integrate genome-wide marker development with fine-scale population analysis in multi-stress coastal wetlands, leaving a critical gap in understanding how genetic diversity buffers compound environmental pressures.

Reed is a pivotal species in wetland ecosystems, with its role being essential for maintaining ecosystem functionality. Nevertheless, there remains a paucity of SSR markers capable of discerning subtle genetic differences among reed populations over relatively confined geographic areas. In light of this shortcoming, the present study employs the whole genome of reed to develop and screen SSR molecular markers. The objectives are to design efficient primers and to validate their reliability and stability in assessing genetic diversity among small-scale reed populations in the Nanhui Dongtan region. This work lays the technical foundation for future efforts aimed at selecting high-quality reed populations that are better adapted to high-salinity environments.

The specific objectives of this study are as follows:

(1) Development and screening of reed SSR primers: To utilize simple sequence repeats derived from the entire reed genome in order to develop and screen SSR primers that provide a robust tool for investigating reed genetic diversity.
(2) Validation of primer stability and reliability: To use the reed community in Shanghai Nanhui Dongtan as a case study to validate the stability and reliability of the newly developed primers, thereby confirming their feasibility.
(3) Assessment of genetic diversity and population differentiation: To employ the developed primers in exploring the genetic diversity and population genetic differentiation of four reed communities in Nanhui Dongtan, thus establishing an important foundation for future selective breeding for salt tolerance and ecological restoration initiatives.

This study has significant scientific and practical implications, such as the development of high-resolution primers into *P. australis* based on its entire genome sequence. These primers enable stable and reliable detection of reed genetic diversity while exploring the effects of salinity gradients on population genetic structure. Furthermore, the research provides a viable basis for the development of SSR markers and actionable insights for designing conservation for reeds in Shanghai Nanhui Dongtan by confirming the genotyping and genetic characteristics of the collected samples, thereby establishing the groundwork for comprehensive evaluations of genetic diversity and genetic structure. Given the global distribution and ecological importance of reeds, a deeper understanding of their population genetics is crucial for devising effective conservation, restoration, and management strategies for coastal wetland restoration—an urgent priority given the accelerating decline in global ecosystems.

## 2. Materials and Methods

### 2.1. Common Reed Samples

**Figure 1:**
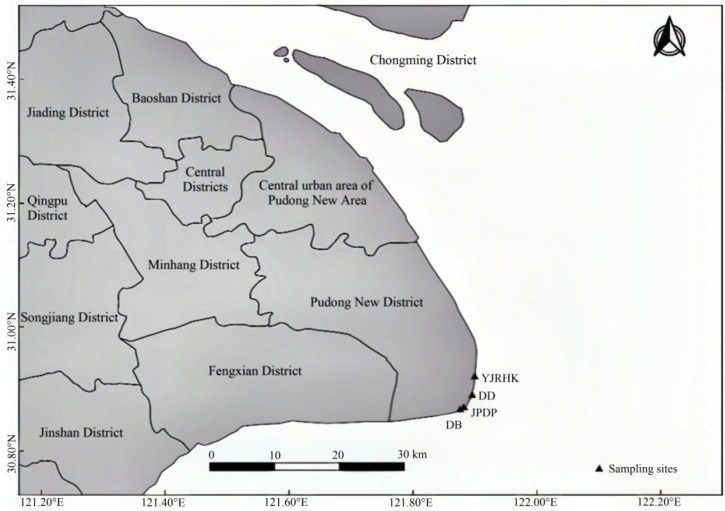
Distribution of sampling sites in Nanhui Dongtan, Shanghai. Base geographic data were obtained from OpenStreetMap (https://wiki.openstreetmap.org/wiki/About_OpenStreetMap accessed on 6 March 2022).

The experimental samples utilized in this study originated from two artificially planted and two wild reed populations in Nanhui Dongtan, Shanghai. The specific location sites for sampling are shown in Figure 1.

### 2.2. Sampling Sites

For the study area of Nanhui Dongtan, four sampling sites were set up (Figure 1; Table 1): inside the embankment (wild population; DD), outside the embankment and inside the open breakwater (planting population; JPDP), outside the embankment and inside the enclosed breakwater (planting population; YJRHK), and shoal outside the embankment (wild population; DD). Each sampling area covered approximately from 2 to 6 hm^2^. Samples were initially categorized and numbered according to geographical distribution. They were photographed and recorded. A 1 m × 1 m sample plot was set up for each population. Plants were collected using the 5-point sampling method, retaining the top layer of inter-root soil in a sampling bag, which was taken back to the laboratory. Thirty samples were collected from each population, amounting to 120 *P. australis* across the 4 populations, and plant height and leaf length were measured. Perform a one-way ANOVA (*p* < 0.05) on the plant height and leaf length of reeds using Prism. Above-ground leaf parts of reeds were taken for DNA extraction and SSR sequencing analysis.

### 2.3. DNA Extraction

Fresh, vigorous and disease-free reed leaves weighing 0.5–1 g from two artificial plantations and two wild reed populations in Nanhui Dongtan, Shanghai, were selected and ground into powder with liquid nitrogen, and DNA was extracted from 120 individual reeds using a TIANGEN DP304 kit, respectively. The purity and concentration of the DNA were tested using an ultraviolet spectrophotometer. After passing the tests, the DNA was uniformly diluted to a concentration of 30 ng·μL^−1^ and stored at −20 °C for further use. After passing the test, the DNA was uniformly diluted to a concentration of 30 ng·μL^−1^ and stored in a refrigerator at −20 °C.

### 2.4. Development of SSR Molecular Markers

The reference genome sequence was downloaded from NCBI for SSR primer screening (GenBank assembly accession: GCA_021018715.1). SSR primers were screened using the MISA (http://pgrc.ikpgatersleben.de/misa accessed on 10 December 2024) according to the criteria that the repeat unit is 3–5 bases and the number of repeats is specified and sufficient. An SSR statistics file (GCA_021018715.1_PauV1_genomic.fna.statistics) was obtained by using MISA, and 72 qualified SSR loci were finally screened, ensuring marker polymorphism.

The products of 1.5% agarose gel electrophoresis were used for universal screening. For the 72 SSR loci screened, 15 corresponding SSR primers were designed and screened. The sequences on both sides of the SSR loci were used as the source of primer sequences for the initial screening, and the primer design system developed by Maipu was used for the primer design of SSR primers [32], as it integrates the Primer3 algorithm with local sequence optimization functions, avoiding primer dimers and nonspecific binding. The 15 pairs of primers with high polymorphism developed by screening were used for microsatellite analysis and PCR amplification of all individuals. The PCR amplification system was as follows: 2× Taq Master Mix 10 μL, 1 μL each of upstream and downstream primers (10 μM), 1 μL of DNA, 8.8 μL of ddH2O, with a total volume of 20 μL. The PCR amplification procedure was as follows: pre-denaturation at 94 °C for 5 min; denaturation at 94 °C for 30 s, annealing at 55 °C for 30 s, and extension at 72 °C for 20 s, for a total of 35 cycles, followed by a final extension at 72 °C for 10 min.

### 2.5. Analysis of Genetic Diversity

#### 2.5.1. Genetic Diversity and Variation Analysis in Natural Populations

GenAlEx 6.51b2 [33] was used to calculate the number of alleles (*Na*), the number of effective alleles (*Ne*), Shannon’s information index (*I*), observed heterozygosity (Ho), expected heterozygosity (*He*), fixation index (*F*), within-population inbreeding coefficient (*F_is_*), population inbreeding coefficient (*F_it_*), population differentiation rate (*F_st_*) and gene flow (Nm), along with molecular analysis of variance (AMOVA) to determine the proportion of genetic variation between and within populations, principal component analysis (PCoA) cluster analysis to assess the correlation between the degree of genetic differentiation and geographic distribution, as well as correlation calculations of the results and the characteristic distances using the Mantel test. This software is specifically designed for population genetic data, supporting AMOVA, Mantel tests, and diversity index calculations, and is highly compatible with SSR data formats.

#### 2.5.2. Analysis of Strain Genetic Structure

PowermarkerV3.25 was used to calculate polymorphism information content (PIC) and Nei’s diversity index (*H*), UPGMA clustering analysis was performed on reed structures, and UPGMA trees were plotted and exported to the iTOL online tool for landscaping and editing [34].

#### 2.5.3. Analysis of Group Structure

To analyze the population structure using STRUCTURE 2.3.4 software, set the K value ranging from 1 to 10, with each K value iterated and recalculated 20 times. Set the burn-in period to 5000 times and the MCMC (Markov Chain Monte Carlo) iterations to 50,000. After compressing the analysis results, import them into the Structure Harvester website (http://taylor0.biology.ucla.edu/structureHarvester/# accessed on 15 December 2024). Based on the principle of maximum likelihood value, plot a curve with K as the horizontal axis and ΔK as the vertical axis. Calculate the peak value of ΔK and select an appropriate K value (the optimal number of populations) at the inflection point. The Bayesian clustering method was adopted, as it can process mixed genotype data and estimate the optimal number of subpopulations, reflecting complex genetic structures more accurately compared to PCA or adjacency methods.

### 2.6. Statistical Analysis of Data

The height and length of the reed (measured from the soil surface to the plant tip) were directly assessed. The raw data files obtained from the online capillary electrophoresis results were imported into Genemarker 4.0. Subsequently, peak plots and SSR locus information tables were exported by locus. Using one primer for each allelic locus, the fragment sizes of each sample at each allelic locus were tabulated into a 0/1 matrix. The data were then statistically analyzed using Excel 2019.

## 3. Results

### 3.1. Development of SSR Molecular Markers in P. australis Populations

SSR types were abundant in the reed genome, with two-nucleotide to six-nucleotide repeat types present (Table 2). A total of 81,593 SSRs were obtained from the sequencing results of the reed, and there was a close relationship between the number of repeats of the core sequences and the length polymorphism of the SSRs. The most repetitive type was the two-base type, accounting for 58.46%, and the hexanucleotide type was the least, accounting for only 0.40%.

This section may be divided by subheadings. It should provide a concise and precise description of the experimental results, their interpretation, as well as the experimental conclusions that can be drawn. In this context, “c” denotes a composite base, while “c*” refers to a multiple composite base, where the asterisk (*) does not require special notation.

Using 1.5% agarose gel electrophoresis, primers with products and obvious main bands were screened. Finally, using 3730XL (Thermo Fisher Scientific. Foster City, CA, USA) capillary electrophoresis screening, 15 pairs of primers with rich polymorphism, good reproducibility and stable amplification of clear bands were obtained and used in formal experiments (Table 3).

### 3.2. SSR Molecular Marker Polymorphism Analysis

After polymorphism analysis of 15 pairs of SSR primers, the results showed that these primers had high PIC indices, with loci LW407 and LW450 having the highest PIC (0.872), and an average PIC index of 0.770 (0.577~0.827), which directly reflected the superiority and stability of the polymorphism of the primers. The 15 pairs of polymorphic primers produced a total of 113.5 *Na*. Each primer pair was mainly generated from ~3.750 to ~13.000 alleles, with an average frequency of 7.567; *Ne* was 4.437, *I* was 1.485, PIC was 0.770, and *H* was 0.794. The above results indicated that the 15 pairs of SSR molecular markers were relatively rich in genetic diversity among the 120 reed germplasm resources collected.

Among them, the observed heterozygosity of the primers was less than the expected heterozygosity (*Ho* < *He*) in only two loci, indicating that there were more heterozygotes in the analyzed loci. 13 loci had negative Fis, ranging from −0.599 to 0.330, with a mean value of −0.223, among which locus LW442 had the largest inbreeding coefficient, with obvious insufficient heterozygosity, a high heterozygosity frequency in general, and more distant crosses in the local population; 11 loci had negative *F_it_*, with a mean value of 0.794; and 11 loci had negative *F_is_*, with a mean value of 0.770, and 0.794. loci had negative *F_it_*, ranging from −0.416 to 0.522, with a mean value of −0.073, indicating a high frequency of heterozygosity, frequent gene exchange, and more distant interbreeding in the whole population; one locus had *F_st_* indicating a very high level of genetic differentiation among the populations (*F_st_* > 0.25), and one locus showed a high level of genetic differentiation among the populations (0.15 < *F_st_* < 0.25), the The rest of the loci indicated a moderate level of genetic differentiation among populations (0.05 < *F_st_* < 0.15), ranging from 0.087 to 0.287, with a mean value of 0.132, and a moderate level of genetic differentiation among various populations of reeds as a whole. There were two loci less than 1 in *Nm*, and the rest were all greater than 1, ranging from 0.622 to 2.615, with a mean value of 1.865, suggesting that each locus reflected a richer genetic exchange among populations (Table 4).

### 3.3. Analysis of Genetic Diversity in 4 Populations

At the population level (Table 5), *Na* for each SSR locus ranged from 2.600 to 12.667, with a mean value of 7.567; *Ne* ranged from 2.231 to 6.640, with a mean value of 3.719; and *I* ranged from 0.817 to 2.020, with a mean value of 1.355 where the reed population with the highest *I* was JPDP, suggesting that genetic diversity was relatively the richest in this population. *Ho* ranged from 0.838 to 0.867, with a mean value of 0.838, and *He* ranged from 0.529 to 0.797, with a mean value of 0.681. Among them, the *Na*, *Ne* and *I* were the largest in the JPDP population and the smallest in the DD population. The *Ho* of all populations was significantly larger than *He* and *F* was negative, indicating that there were more heterozygous individuals in the various populations of the group. The above results showed the genetic diversity of different populations of reed to a certain extent, indicating that the overall genetic diversity level of reed populations was high.

The results of the AMOVA analysis (Table 6) showed that 12% of the variation came from among populations, indicating that there was no obvious heterogeneity among reed populations, which belonged to the lower level of genetic differentiation, and 88% of the variation came from among individuals, which was the main source of variation, which was consistent with the results of the analysis of the genetic differentiation of reed populations and the study of the genetic diversity of reed populations.

**Figure 2:**
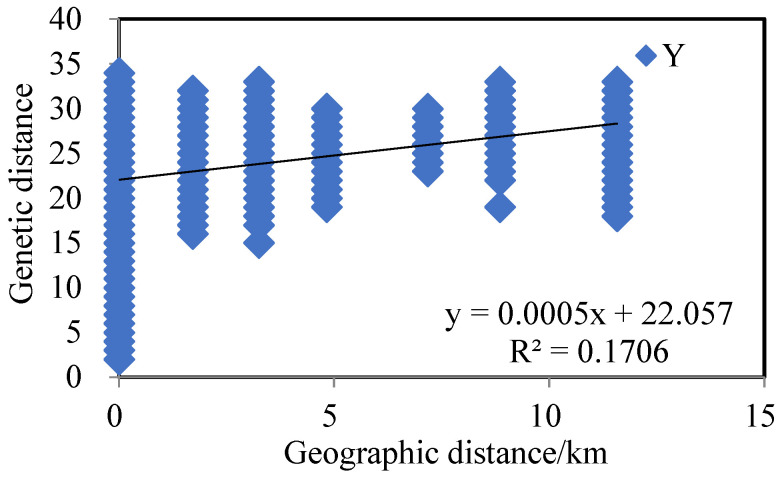
Correlation between genetic distance and geographical distance of 4 Phragmites australis populations.

**Figure 3:**
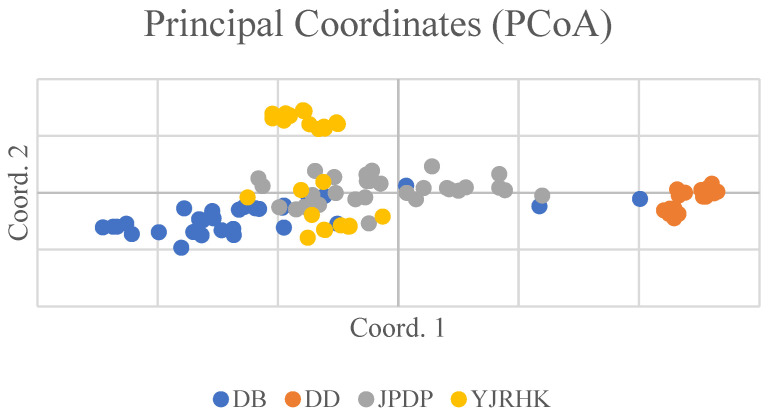
Principal coordinate analysis (PCoA) of Phragmites australis germplasm resources based on 15 SSR markers.

The Mantel test was conducted on the genetic distance matrix and the geographic distance matrix among the four populations, with calculations based on 999 random permutations. The genetic distances of the four populations of reed selected for this experiment were correlated with the geographic distances (Figure 2), y = 0.0005x + 22.057, and the R^2^ value of the correlation between genetic distances and geographic genetic distances was 0.1706, with a significance level of *p*-value of 0.001. The above results showed that the geographic distribution of the reed populations and genetic distances showed a significant link between them and that the regions with similar distances had genetic similarities. According to the results of principal component analysis (PCoA) (Figure 3), PCoA1 and PCoA2 accounted for 20.48% and 12.44% of the total genetic variation in all reed germplasm, respectively.

### 3.4. Analysis of the Genetic Structure of 4 P. australis Populations

GenAlEx 6.51b2 software was used to obtain Nei’s genetic identity (GI) and genetic distance (GD) data among four reed populations with different geographic origins, and the results showed (Table 7) that Nei’s genetic identity (GI) among the populations ranged from 0.401 to 0.745, with a mean value of 0.595, and Nei’s genetic distance (GD) ranged from 0.294 to 0.915, with a mean value of 0.547, with the smallest genetic distance (GD = 0.294) and the largest corresponding genetic consistency (GI = 0.745) between populations of DB and JPDP, which had more similar genetic backgrounds, and the genetic distance between populations of DD and YJRHK was the largest (GD = 0.915) and the corresponding genetic identity was the smallest (GI = 0.401).

**Figure 4:**
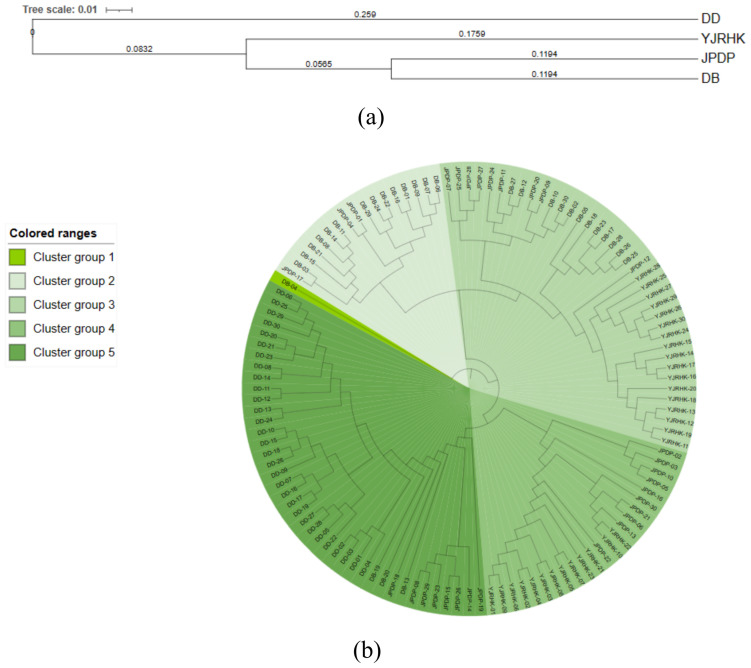
UPGMA dendrogram for (**a**) populations of Phragmites australis based on Nei’s genetic distance and (**b**) individuals of *P. australis* based on Nei’s genetic distance.

UPGMA clustering was performed on 4 reed populations (Figure 4a) and 120 reed DNA samples (Figure 4b) according to Nei’s genetic distances. The 4 reed populations and 120 reed DNA samples were classified into two major subgroups and five clusters. The analyses showed that the first subgroup of the UPGMA clustering diagram, i.e., cluster 1, included only one individual from the DB population, and the majority of the UPGMA clustering diagram consisted of the second largest subgroups (containing two to five clusters), with cluster 2 containing a total of 17 individuals, including 14 individuals from the DB population and three individuals from the JPDP population, and cluster 3 containing a total of 38 individuals, including 12 individuals from the DB population, nine individuals from the JPDP population, nine individuals from the YJRHK population, and three individuals from the YJRHK population. 9 plants from the DB population, 17 plants from the YJRHK population; the 4th cluster had a total of 23 individuals, including 10 plants from the JPDP population and 13 plants from the YJRHK population; and the 5th cluster had a total of 41 individuals, including 3 plants from the DB population, 30 plants from the DD population, and 8 plants from the JPDP population.

**Figure 5:**
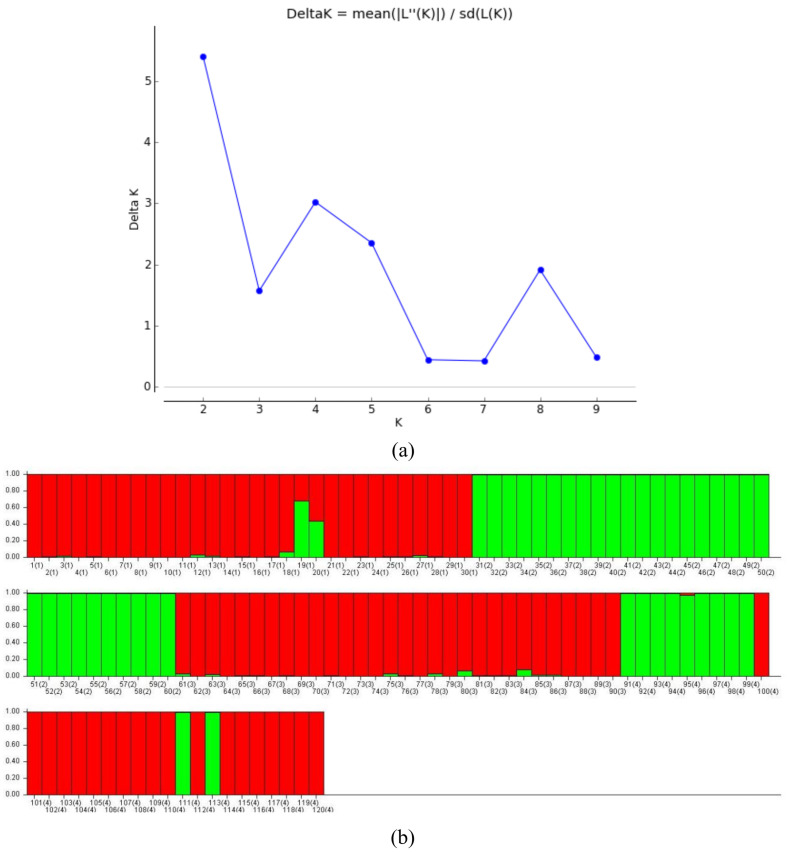
(**a**) Estimated values of L’(K) and ∆K on structure analysis and (**b**) structure clustering analysis (K = 2).

To reveal the population structure of 120 reed DNA samples, the genetic structure of four population groups from different geographic distributions was analyzed based on a Bayesian approach, where the putative population size (gene pool) was expressed as the K value. Based on the results of the K versus ΔK line plot, it was found that the log-likelihood function value L’(K) showed a maximum peak in ΔK when K = 2 (Figure 5a), indicating that the suitable subpopulation number of *P. australis* is 2.

The natural population was analyzed for population genetic structure using Structure 2.3.4 software and Q values were calculated for each material (Figure 5b). The number of materials in the two subpopulations was 78 and 42, respectively, with subpopulation 1 (marked in red) containing 29 copies of the DB population, 30 copies of the JPDP, and 19 copies of the YJRHK; and subpopulation 2 (marked in green) containing 1 copy of the DB population, 30 copies of the DD population, and 11 copies of the YJRHK.

Of the 120 reeds, 116 (96.7%) had a Q ≥ 0.6, which is presumed to be a relatively homogeneous genetic component, and 4 individuals (3.3%) had a Q < 0.6, which is a more complex genetic background: DB3-19 (19), DB3-20 (20), YJRHK-11 (111), and YJRHK-13 (113).

### 3.5. Selection of P. australis Populations with Salinity

In CGE analysis, applying diverse primers to different populations yields distinct peak patterns with variations (Table 8). Specifically, the JPDP population exhibits a rich diversity of peak patterns (12–24 types), while the DB population has fewer types (7–12). The YJRHK population further limits peak pattern variety (2–4 types, mostly 3), and the DD population maintains a uniform pattern (one type). From a molecular biology perspective, individuals with identical peak patterns belong to the same group. Using this criterion, the DD population has the smallest subset of subgroups, indicating low genetic diversity, whereas the JPDP group shows the highest species diversity. Among the 30 plants in the DD population, which exhibit competitive potential against *S. alterniflora*, only one type of peak was observed in capillary electrophoresis of microsatellite loci. In contrast, plants from three other populations exhibited more than three types of peaks. This finding suggests that the DD population is undergoing natural selection in an environment with higher salinity stress.

**Figure 6:**
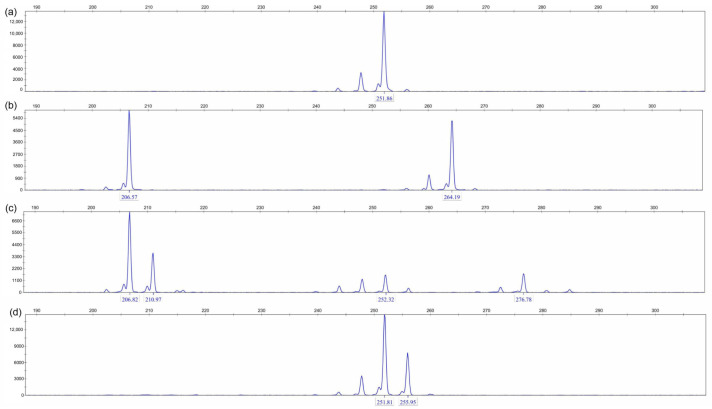
The diversity of amplification using the LW401 primer across four DNA samples: The figure shows CGE partial results for the LW401 primer from (**a**) DB, (**b**) DD, (**c**) JPDP, and (**d**) YJRHK.

Among the 30 plants in the DD population, which exhibit competitive potential, only one type of peak was observed in capillary electrophoresis of microsatellite loci (Table 8; Figure 6). In contrast, plants from three other populations exhibited more than three types of peaks. This finding indicates that the DD population is undergoing natural selection in an environment characterized by severe salinity stress, effectively functioning as a sieve for salt-tolerant individuals.

Salinity stress plays a role in screening and purifying populations. Specifically, there exists a negative correlation between the intensity of salinity stress and the uniformity of PCR product peak patterns: the more intense the salinity stress, the more uniform the PCR product peak patterns tend to be, indicating a simpler population structure; conversely, the less intense the salinity stress, the more diversified the peak patterns appear, reflecting an increased complexity in population structure.

### 3.6. Distribution and Epigenetic Characteristics of Major P. australis Populations in Typical Coastal Wetlands of Nanhui Dongtan, Shanghai

There are four mainly reed populations distributed in the typical coastal wetland of Nanhui Dongtan, Shanghai (Table 9): (1) the marshy zone within the dyke, extending along the bank of the river ditch, shallow water marshes, and the slopes of artificial hillocks, with the salinity of the water body of 4–10 ppt, and the distribution of reeds covering an area of about 2.1 hm^2^; (2) the upland zone outside the dyke, with the elevation of 3.5–6.0 m, which is accessible to the seawater in high tides, and the salinity of the seawater is 7–12 ppt, soil salinity and alkalinity are high, reed area is about 4.5 hm^2^; (3) enclosed mudflat, outside the dyke and on the mudflat enclosed by a large area and is affected by tides daily, seawater salinity is 7–12 ppt, reed area is about 6.2 hm^2^; (4) open mudflat, outside the dyke and affected by the tides daily, seawater salinity is 7–12 ppt, 2022 reed area is approximately 3.4 hm^2^.

The DB populations remained stable and unaffected by wind, waves, and tides in their immediate vicinity. In stark contrast, both the JPDP and YJRHK populations experienced significant impacts from wind, waves, and tides. However, the YJRHK population experienced relatively less wind and wave impact compared to the JPDP population. Conversely, the DD population faced the strongest wind and wave impacts due to the absence of berm protection. This specific area was initially occupied by *S. alterniflora*, with reeds eventually emerging and growing amidst it.

**Figure 7:**
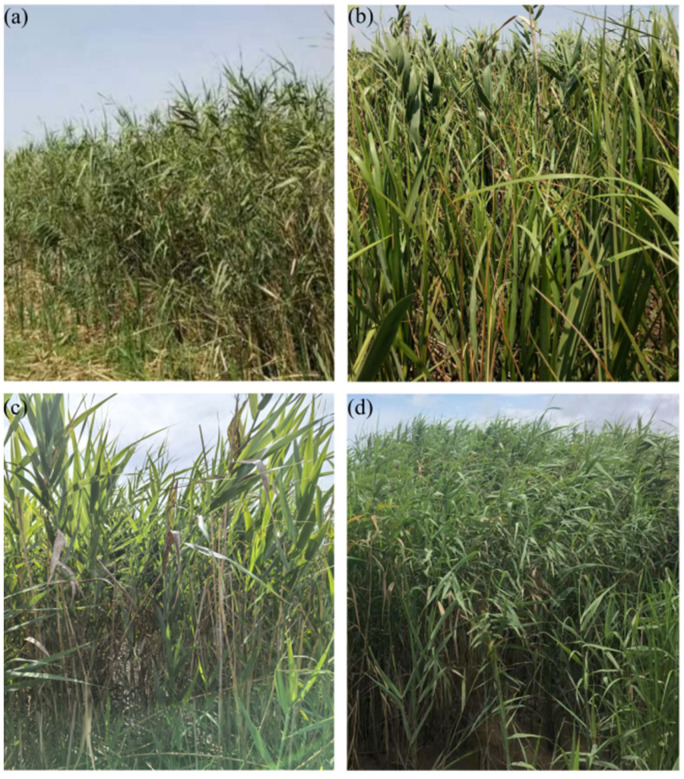
Phragmites australis population in Nanhui Dongtan, Shanghai: (**a**) DB; (**b**) JPDP; (**c**) YJRHK; (**d**) DD.

Samples were collected from one location in each of the four designated population distribution areas to compare the epigenetic characteristics of four distinct *P. australis* populations (Figure 7).

**Figure 8:**
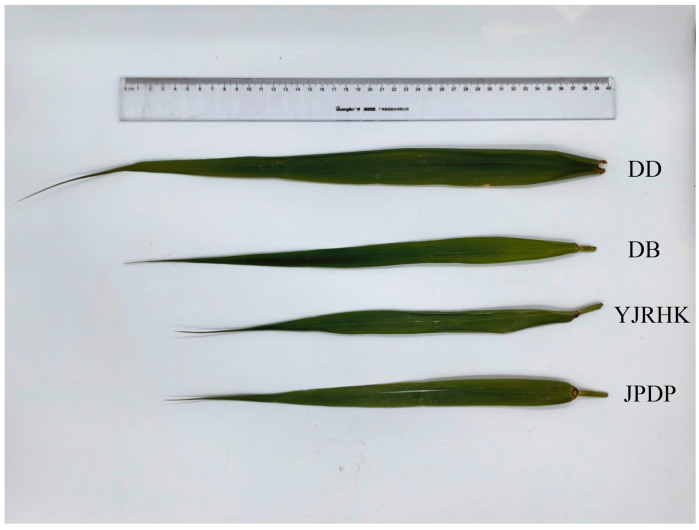
Epigenetic differences in leaf morphology among four Phragmites australis populations: From top to bottom, the leaf morphologies belong to DD, DB, YJRHK, and JPDP, respectively.

The field survey revealed some differences in morphological characteristics among the four populations, with plant height being the most pronounced, with the highest height in the DD population and the lowest in the DB population (Table 10). Comparisons revealed significant morphological differences between reed blades from the four different growth locations (Figure 8). Reed leaves from the mudflat outside the dyke were the longest of the four, with bright green color, intact tips and well-defined veins. DB leaves were of medium length but slightly smaller compared to mudflat leaves, probably because the interior of the dyke, although stable, was still somewhat limited compared to the mudflat. YJRHK leaves were short in length, slightly lighter in color, and overall thinner and narrower. JPDP leaves were the shortest, palest in color, and with relatively faint tips and veins.

## 4. Discussion

### 4.1. Population Genetic Diversity and Differentiation

In this study, SSR analysis was conducted on 120 samples from four reed populations (DB, JPDP, YJRHK, and DD) collected from Nanhui Dongtan, Shanghai. The results indicate that the overall genetic diversity is high, while moderate genetic differentiation is evident among populations. Examination of diversity parameters (refer to Table 7) reveals that the mean number of alleles (*Na*) is 7.567, the effective number of alleles (*Ne*) is 4.437, and the Shannon diversity index (*I*) reaches 1.485. With observed heterozygosity (*Ho*) at 0.853 versus an expected heterozygosity (*He*) of 0.690, these findings demonstrate that reed populations in coastal environments have accumulated substantial genetic variation. Notably, the JPDP population exhibits the highest *Na* and *I* values (*Na* = 12.667; *I* = 2.020), whereas the DD population records the lowest values for both metrics. This gradation in genetic diversity not only highlights inter-population differences but also suggests that the JPDP population may have experienced more frequent gene exchange or the introduction of external material, whereas the DD population, confronted with pronounced salt stress and competition from *S. alterniflora*, appears to have undergone marked allele loss or directional selection, thereby reducing its overall diversity.

From an ecological perspective, higher genetic variation is generally associated with greater adaptive capacity [16]. The Nanhui Dongtan region, characterized by complex tidal dynamics, fluctuating salinity levels, and anthropogenic disturbances, appears to have driven the retention or accumulation of beneficial alleles related to salt tolerance, wave resistance, and rhizome architecture [19]. The detection of elevated genetic diversity under these multifaceted environmental pressures reinforces the notion that reeds possess considerable ecological adaptability [35,36]. Moreover, it provides crucial insights into the synergistic roles of salinity and wave impact in shaping population structure.

Regarding genetic differentiation, the results obtained from *F_st_* estimates, AMOVA, UPGMA clustering, and structure analyses collectively reveal a moderate level of differentiation among populations. The average *F_st_* is 0.132, and the AMOVA indicates that approximately 12% of the total genetic variance is attributable to differences among populations, while 88% lies within populations. This pattern suggests that most genetic variation is maintained at the individual level. Furthermore, both UPGMA and structure analyses partition the four geographically distinct populations into two major subgroups—one combining the DD and YJRHK populations, and the other grouping DB with JPDP. Such a pattern reflects the influence of geographical proximity and environmental factors (such as salinity and hydrological conditions) on population structure, while also indicating the persistence of gene flow among populations. The Mantel test further supports this view by demonstrating a significant correlation between geographic distance and genetic differentiation, inferring that both spatial isolation and environmental gradients characteristic of coastal wetlands jointly drive the observed patterns of population differentiation over extended timescales—a factor critical to understanding the evolutionary trajectory of these populations [37,38].

Moreover, analyses based on SSR markers (including PCoA and UPGMA clustering) confirm that, although the four populations are evolutionarily distinct, there is evidence of considerable genetic admixture [39]. Reed populations, even in extreme or highly disturbed environments, exhibit robust survival and dispersal capabilities accompanied by notable plasticity across variable habitats [40,41]. For example, the JPDP population—shaped by an interplay of anthropogenic and natural influences—displays a rich allelic composition and marked admixture, suggestive of extensive gene flow. Taken together with findings from other regions, these results imply that reeds can maintain substantial local diversity under various environmental pressures [42]. Rapid gene flow, coupled with adaptive evolution, enables reed populations to effectively respond to fluctuating ecological conditions. Thus, the extent of population differentiation observed reflects the dual influence of environmental heterogeneity and the species’ inherent evolutionary potential.

In summary, the reed populations in Nanhui Dongtan exhibit high overall genetic diversity coupled with moderate differentiation. Significant population structure differences can be observed even over relatively small geographic scales. The exceptionally high diversity observed in the JPDP population, contrasted with clear signals of directional selection in the DD population, provides a robust foundation for subsequent efforts in adaptive screening and ecological restoration. The SSR markers developed herein offer valuable tools for elucidating the microevolutionary processes and adaptive mechanisms underlying the reed’s response to environmental stress.

### 4.2. SSR Genetic Diversity of Reed

Genetic diversity encompasses the entire set of genetic information present within a biological population. It serves as a fundamental basis for biodiversity assessment and is a prerequisite for species to adapt to variable environmental conditions, ensuring long-term survival and evolution [43]. Previous studies have indicated that reed exhibits moderate genetic diversity, characterized by extensive gene flow among populations and a predominance of genetic variation at the individual level. This pattern may arise from differential genotype expression under varying environmental conditions—a finding that has also been observed in studies of two reed subspecies using eight SSR markers in Wisconsin [30].

In the present study, fifteen novel SSR primer pairs were developed and screened based on the whole-genome sequence of *P. australis*. These primers demonstrated high levels of polymorphism and robust amplification consistency. Capillary electrophoresis analysis revealed an average number of alleles (*Na*) of 7.567 per locus, with the allele count per locus ranging from 3.750 to 13.000, effectively capturing the genetic variation among individual plants. The Shannon diversity index (*I*) across the four source populations ranged from 0.904 to 2.008, with an average of 1.485, indicating that the loci provided a rich reservoir of genetic information for characterizing population-level diversity. These values are significantly higher than the polymorphism observed in reed populations in small river habitats in Lithuania, even between stations within the same river (ISSR, *I* = 0.299 ± 0.028), which may be related to the more complex environment of tidal flats compared to river environments [39].

Furthermore, *P. australis*’s adaptive strategies align with but also diverge from mechanisms observed in other wetland plants. For instance, after the invasion of *S. alterniflora* into the growth areas of *Suaeda salsa*, its underground biodiversity has suffered significant impacts, specifically manifested in a continuous decline in the Shannon diversity index. Meanwhile, the content of soil organic carbon exhibits an upward trend, while the concentration of ammonium nitrogen decreases [44]. In contrast, *Juncus effusus* accumulates osmolytes like proline under salinity, a mechanism less pronounced in *P. australis*. Such comparisons underscore the diversity of adaptive pathways in wetland plants, with *P. australis* uniquely balancing genetic diversity and epigenetic plasticity [45].

Gene flow (*Nm*) is a critical factor influencing the degree of genetic differentiation among species and communities [46]. Generally, *Nm* values below 0.250 indicate low gene exchange, values between 0.250 and 1.000 suggest moderate exchange and values exceeding 1.000 denote high levels of gene flow [47]. In this study, the average *Nm* was 1.865, signifying substantial gene exchange between populations. Such high gene flow can mitigate the effects of inbreeding, preventing pronounced genetic drift and differentiation. The observed moderate inter-population differentiation is thus likely influenced by frequent gene flow, while the combined effects of geographical location and environmental gradients also play a significant role [48,49]. For instance, populations spanning large geographic distances (such as those from the middle-lower Yangtze region to the Yungui Plateau) exhibit *Nm* values around 0.215, reflecting marked genetic differentiation [50].

The polymorphism information content (PIC) of the SSR markers averaged 0.770, with individual values ranging from 0.577 to 0.872, thereby confirming a high level of marker polymorphism and strong discriminatory power. Observed heterozygosity (*Ho*) and expected heterozygosity (*He*) were also high across most loci, with average values of 0.853 and 0.690, respectively, indicating an abundance of heterozygous genotypes within the populations. Notably, aside from a few loci indicating heterozygote deficiency (*Ho* < *He*), the majority of loci displayed negative overall inbreeding coefficients (*F*) and within-population inbreeding coefficients (*F_is_*), which suggests a prevailing outcrossing tendency. This pattern is likely attributable to the wide geographic distribution of reed, continuous gene flow among populations inhabiting different environments, and the cumulative effects of long-term natural selection.

Additionally, chloroplast genomic microsatellite markers can not only be used to identify plant species but also reveal the genetic relationship and genetic structure among different plant populations [51]. Chloroplast rpl16 and other genes were employed to clarify the delineation and evolutionary relationship among four *P. australis* ecotypes in the Hexi Corridor [52]. Saltonstall [3] used SSR markers based on microsatellite enrichment technology to discover three *P. australis* strains of North America and Europe and introduced species hybridized with genetic differences. And further investigated the hybridization phenomenon between native North American and introduced European *P. australis*. Yu et al. [53] constructed a microsatellite-enriched library, designed six polymorphic microsatellite loci for reeds, and validated their polymorphism using reeds from Chongming Island in Shanghai and Qi’ao Island in Zhuhai.

In addition to high polymorphism, the fifteen SSR primer pairs exhibited excellent stability. Both agarose gel electrophoresis and capillary electrophoresis consistently produced clear, reproducible amplification bands, thereby avoiding issues such as ambiguous or missing bands. The allele patterns detected at each locus were similar across different samples, and the reproducibility across repeated experiments was high. These attributes establish a strong technical foundation for the widespread application of these markers across diverse regions and larger sets of reed samples.

In conclusion, the SSR markers developed in this study exhibit superior levels of both polymorphism and stability. They not only enable the discrimination of reed populations from different geographic origins but also reveal subtle genetic differences among individuals. The genetic distances observed among the four reed populations in Nanhui Dongtan ranged from 0.294 to 0.915, a range considerably higher than the 0–0.05 interval commonly reported among local populations [54], and notably larger than the 0.2362–0.3443 range reported for four ecotypes in another region [42]. These findings reflect the enhanced impacts of geographic location, hydrological conditions, and salt stress on genetic differentiation. In conjunction with the AMOVA results—which showed that 12% of the genetic variation resided among populations and 88% within populations—the reliability and versatility of these SSR markers provide robust support for further molecular ecological studies, germplasm resource evaluations, and ecological restoration efforts involving reed.

### 4.3. Applicability and Prospects of SSR Markers

This study developed and screened 15 SSR markers based on whole-genome information, demonstrating their reliable polymorphism and robust amplification stability in *P. australis*. Compared with previous studies that employed random primers or microsatellite enrichment methods, our SSR markers provide superior resolution in detecting allele numbers, polymorphism information content (PIC), and genetic differentiation. They are particularly sensitive in capturing population differences driven by subtle environmental variations such as salinity gradients, wave disturbances, and anthropogenic land-use practices.

Owing to their operational simplicity, low cost, high reproducibility, and broad applicability, these SSR markers are well suited not only for germplasm surveys and conservation efforts in coastal wetland reed populations but also for genetic structure analyses in other wetland types, riparian zones, and cultivated areas.

Utilizing these SSR markers across diverse ecological environments facilitates the elucidation of the adaptive and evolutionary trajectories of *P. australis* under multifaceted pressures—including salt stress, tidal fluctuations, and human disturbances. Coastal wetlands typically experience several concurrent stressors (e.g., pronounced salinity gradients, variable sediment deposition rates, and infrastructural modifications), which may induce directional selection or genetic drift in reed populations. By achieving high-resolution monitoring of genetic diversity parameters (such as allele numbers, genetic distances, and *F_st_* values), these markers enable the identification of genotypes with enhanced resilience to adverse conditions, thereby providing a scientific basis for precise ecological restoration and resource management strategies.

Furthermore, under varying remediation practices and land-use scenarios (e.g., saline-alkali land improvement, tidal channel dredging, or control measures against invasive *S. alterniflora*), these markers can be used to continuously monitor gene flow, introgression events, and potential population contraction or expansion. Integrating such molecular data with physiological phenotypes (e.g., osmotic regulator levels, stomatal characteristics, and rhizome development metrics) and epigenetic indicators (e.g., DNA methylation profiles, histone modification patterns) can form a solid foundation for breeding reed varieties that combine salt tolerance, waterlogging resistance, and high biomass production. With the advent of phenomics and integrative omics analyses, SSR markers offer distinct advantages for positional mapping and core germplasm selection, ultimately facilitating the evolution of reed from a passive ecological restoration tool into a proactive “ecological engineering material”.

Importantly, these highly polymorphic and operationally robust SSR markers are not only applicable for investigating *P. australis* adaptive patterns along environmental gradients but also hold promise for cross-species comparative studies involving closely related genera or other herbaceous wetland species. Wetland plant communities are characterized by complex interspecific relationships, and SSR-based analyses can help unravel the intricacies of gene flow, genetic structure reorganization, and competitive strategies—especially in scenarios involving invasive species. For example, comparative population genetic studies between reed and dominant or invasive species such as *S. alterniflora* can illuminate the core microevolutionary mechanisms underlying plant invasions or species displacement, and can further enhance our understanding of rhizosphere microbial interactions and epigenetic plasticity across species.

The contrasting genetic diversity between DD and JPDP populations may reflect divergent reproductive strategies under environmental stress. While DD likely prioritizes clonal expansion for rapid habitat occupation under saline competition, high heterozygosity of JPDP suggests active sexual recruitment, possibly facilitated by human-mediated seed introduction during restoration. Such a trade-off between clonal persistence and sexual adaptability has been widely observed in wetland plants facing heterogeneous stressors.

Moreover, with ongoing advancements in high-throughput sequencing and the rise of emerging disciplines like phenomics, the SSR markers developed in this study can be integrated with other molecular techniques (e.g., AFLP, SNP arrays, and transcriptome sequencing) to establish a cross-platform, comprehensive analytical framework. Such an integrative approach will significantly strengthen investigations into the dynamic evolution and adaptive mechanisms not only of *P. australis* but also of entire wetland plant communities. Given the critical role of reeds in maintaining wetland biodiversity and ecosystem function, these multi-source molecular data provide precise scientific guidance for ecological restoration planning, invasive species control, and the implementation of sustainable management strategies.

In summary, the SSR markers developed in this study exhibit high applicability and stability, making them exceptionally promising for future research in wetland botany, ecology, and conservation genetics.

## 5. Conclusions

This study established 15 polymorphic SSR markers from the whole-genome sequences of *P. australis* and used them to evaluate the genetic diversity and population structure of four distinct reed populations in Nanhui Dongtan, Shanghai. Analyses revealed high genetic variation, mainly within populations, as AMOVA indicated. Despite shared diversity, populations formed two major subgroups due to environmental selection, geographic distance, and gene flow. Notably, the JPDP population possessed the greatest diversity, suggesting ongoing genetic influx, while the DD population—subjected to salt stress and interspecific competition—exhibited the lowest genetic diversity. These results underscore the significant adaptive potential of *P. australis* in dynamic coastal wetlands and its vulnerability to specific environmental pressures.

From an applied perspective, the SSR markers are highly reliable and discriminatory, suitable for further ecological, genetic, and evolutionary investigations of *P. australis* under heterogeneous environmental gradients. They also aid wetland restoration initiatives and guide the selection of salt-tolerant, high-biomass germplasm. In conclusion, our SSR markers reveal microevolutionary processes in coastal ecosystems and provide essential molecular tools for conservation, ecological restoration, and future breeding endeavors in salinity-prone wetlands.

## Figures and Tables

**Table 1:** Source information of 4 Phragmites australis populations.

Sampling Sites	Codes	Longitude	Latitude	Altitude
Inside the embankment (wild population)	DB	121°53′46″ E	30°51′18″ N	2.03 m
Outside the embankment and inside open breakwater (planting population)	JPDP	121°52′46″ E	30°50′58″ N	3.02 m
Outside the embankment and inside enclosed breakwater (planting population)	YJRHK	121°58′23″ E	30°54′56″ N	2.40 m
Shoal outside the embankment (wild population)	DD	121°55′36″ E	30°51′53″ N	1.82 m

**Table 2:** Number and distribution of SSRs based on the genome of Phragmites australis.

Repetition Type	Total	Proportion/%	Repetitions										
			5	6	7	8	9	10	11	12	13	14	≥15
p2	47,697	58.46	0	15,011	7600	4963	3284	2010	1335	1080	887	797	10,740
p3	21,876	26.81	14,487	4534	1532	568	254	106	85	58	41	37	174
p4	2204	2.70	1590	335	115	55	23	13	10	18	5	2	38
p5	651	0.80	487	123	30	9	1	0	1	0	0	0	0
p6	326	0.40	245	55	7	2	5	2	2	0	1	0	7
c	8440	10.34	0	0	0	0	0	35	30	101	103	114	8057
c *	399	0.49	0	0	0	0	0	3	17	16	23	22	318

**Table 3:** Information of 15 pairs of SSR primers.

Locus	Primer Sequences	Repeating Units	Number of Bases	Range/bp
LW401	F:GGAATGCTACTGTATTAATGTCGTT R:GTACCACCAAGATGCCCTCA	TGTA	4	206–272
LW407	F:TTAGGACGAGAGCAAGAAGCC R:CGACTGGGACAGAGGAACG	GAG	3	288–315
LW423	F:GCATCAGTCTCCTTGTACCGTT R:GTTACTCCTTCGCCGACACTT	TTG	3	292–311
LW424	F:GCGTCGAGTCGTTTGAAACC R:CATCACCAGCGACCTCCG	GAG	3	358–391
LW442	F:ATTACAACTTCTCGCTTCGGAT R:CAGTTGACATTCCATTTCCAGC	ATAC	4	217–229
LW444	F:ACAAGAGGCTGAAACGAACG R:CGCATGACGAACCAATAACA	AAG	3	268–329
LW450	F:TTGGATACACCATCATTGTTCATAG R:GCTGCTGACGGTCAACCTT	TATG	4	282–407
LW459	F:TTGGCTGATGGGAAGTTGTCT R:GGAGCAGAATCAAAGCAGGC	TGC	3	310–325
LW467	F:GCAACTGAACTGGGAAGACAAC R:TTCAAGTGGAGCAGTATCGTCAT	CTAT	4	249–297
LW474	F:AGTCTCTCAGTGTAACCTTCCCA R:GTTGTTATTCTTGCTGATGTGTATTC	GAA	3	266–321
LW479	F:CCTTCTGACTTACTGTCAAGCTCTC R:AGACCCAACTCACCAGGAAAG	TTC	3	268–335
LW482	F:TCAACCACGCATTCGGTG R:CGCAAGGGACAACAGAGGG	CTT	3	178–221
LW542	F:GCCAATCAGCCACACAACG R:CCAGTACGTTCCTTGACCTTG	CCT	3	345–370
LW543	F:TGCTCAGATGTCAGCCAGTTG R:AGCACTTAAAGCAGCGATTGAC	CTT	3	320–381
LW549	F:CCTTGCCAACTTGTCCCAG R:AGATAGGCATTCACGCAAGT	ATAC	4	254–292

**Table 4:** Polymorphic analysis of the 15 SSR markers.

Locus	*Na*	*Ne*	*I*	*PIC*	*Ho*	*He*	*H*	*F*	*F_is_*	*F_it_*	*F_st_*	*Nm*
LW401	9.500	5.368	1.737	0.845	0.975	0.761	0.859	−0.333	−0.282	−0.135	0.115	1.932
LW407	8.000	5.235	1.778	0.872	0.967	0.798	0.883	−0.218	−0.211	−0.094	0.097	2.341
LW423	5.500	3.041	1.185	0.657	0.650	0.621	0.704	0.001	−0.046	0.077	0.117	1.879
LW424	4.250	2.641	1.088	0.639	0.983	0.615	0.694	−0.608	−0.599	−0.416	0.114	1.938
LW442	4.250	2.268	0.904	0.659	0.333	0.497	0.697	0.378	0.330	0.522	0.287	0.622
LW444	7.500	4.508	1.443	0.847	0.517	0.644	0.860	0.347	0.198	0.397	0.248	0.757
LW450	13.000	8.265	2.008	0.872	1.000	0.787	0.880	−0.353	−0.271	−0.137	0.105	2.121
LW459	6.750	3.737	1.458	0.778	0.992	0.718	0.800	−0.394	−0.382	−0.240	0.103	2.183
LW467	10.250	6.040	1.781	0.833	0.983	0.748	0.845	−0.394	−0.315	−0.164	0.115	1.929
LW474	9.250	6.645	1.784	0.858	1.000	0.766	0.870	−0.383	−0.305	−0.149	0.119	1.843
LW479	9.250	4.690	1.658	0.852	0.917	0.740	0.864	−0.315	−0.239	−0.061	0.144	1.486
LW482	3.750	2.384	0.955	0.577	0.633	0.558	0.637	−0.220	−0.136	0.006	0.125	1.755
LW542	7.250	3.327	1.430	0.719	0.867	0.669	0.737	−0.301	−0.295	−0.177	0.091	2.485
LW543	9.000	5.376	1.776	0.865	1.000	0.782	0.875	−0.303	−0.279	−0.142	0.107	2.086
LW549	6.000	3.026	1.286	0.681	0.983	0.648	0.710	−0.556	−0.517	−0.384	0.087	2.615
Mean	7.567	4.437	1.485	0.770	0.853	0.690	0.794	−0.243	−0.223	−0.073	0.132	1.865

**Table 5:** Genetic diversity parameters of Phragmites australis.

Population	*Na*	*Ne*	*I*	*Ho*	*He*	*F*
DB	9.400	5.157	1.747	0.858	0.754	−0.099
DD	2.600	2.231	0.817	0.867	0.529	−0.588
JPDP	12.667	6.640	2.020	0.851	0.797	−0.075
YJRHK	5.600	3.719	1.355	0.838	0.681	−0.212
Mean	7.567	4.437	1.485	0.853	0.690	−0.243

**Table 6:** AMOVA analysis for genetic variation in Phragmites australis.

Source	Degrees of Freedom	Sum of Squares	Mean Square	Variance Component	Ratio of Variance
Among populations	3.000	167.975	55.992	0.852	12%
Among individuals within populations	116.000	564.017	4.862	0.000	0%
Within individuals	120.000	768.000	6.400	6.400	88%
Total	239.000	1499.992	-	7.252	100%

**Table 7:** Nei’s genetic identity and genetic distance of Phragmites australis populations.

	DB	DD	JPDP	YJRHK
DB	-	0.443	0.745	0.693
DD	0.813	-	0.572	0.401
JPDP	0.294	0.558	-	0.717
YJRHK	0.367	0.915	0.333	-

Nei’s genetic identity (GI, on the diagonal) and genetic distance (GD, off the diagonal).

**Table 8:** The number and types of peak patterns amplified by 15 pairs of primers in four populations of Phragmites australis.

Locus	DB	DD	JPDP	YJRHK
LW401	8	1	23	3
LW407	15	1	21	3
LW423	10	1	16	2
LW424	10	1	23	3
LW442	4	1	14	3
LW444	11	1	18	3
LW450	9	1	22	3
LW459	10	1	19	3
LW467	12	1	21	3
LW474	10	1	21	4
LW479	8	1	20	3
LW482	7	1	12	3
LW542	10	1	18	3
LW543	8	1	24	3
LW549	9	1	23	3

**Table 9:** Distribution characteristics of major Phragmites australis populations in typical coastal wetlands in Nanhui Dongtan.

Code	Seawater Salinity/ppt	Area/hm^2^	Wind Waves	Tides
DB	4–10	2.1	none	none
JPDP	7–12	4.5	present	present
YJRHK	7–12	6.2	present	present
DD	7–12	3.4	present	present

**Table 10:** Morphological differences between 4 typical coastal wetlands Phragmites australis populations.

Morphological Characteristics	DB	JPDP	YJRHK	DD
Height/cm	178.1 ± 1.9 *	188.7 ± 2.2 *	204.2 ± 2.4 *	234.9 ± 3.2 *
Leaf length/cm	29.2 ± 0.3 *	36.4 ± 0.3 *	31.7 ± 0.3 *	43.3 ± 0.3 *
Shape of leaves	Lanceolate, flattened, pendulous, finer	Lanceolate, flattened, pendulous, finer	Lanceolate, flattened, pendulous, finer	flattened, pendulous, finer

* Asterisks (*) indicate significant differences at the *p* < 0.05 level.

## Data Availability

Data will be made available on request.
